# Imprinted genes show unique patterns of sequence conservation

**DOI:** 10.1186/1471-2164-11-649

**Published:** 2010-11-22

**Authors:** Barbara Hutter, Matthias Bieg, Volkhard Helms, Martina Paulsen

**Affiliations:** 1 Lehrstuhl für Computational Biology, Universität des Saarlandes, Postfach 151150, D-66041 Saarbrücken, Germany; 2 Lehrstuhl für Genetik/Epigenetik, Universität des Saarlandes, Postfach 151150, D-66041 Saarbrücken, Germany; 3 Theoretische Bioinformatik (B080), Deutsches Krebsforschungszentrum, Im Neuenheimer Feld 580, D-69120 Heidelberg, Germany

## Abstract

**Background:**

Genomic imprinting is an evolutionary conserved mechanism of epigenetic gene regulation in placental mammals that results in silencing of one of the parental alleles. In order to decipher interactions between allele-specific DNA methylation of imprinted genes and evolutionary conservation, we performed a genome-wide comparative investigation of genomic sequences and highly conserved elements of imprinted genes in human and mouse.

**Results:**

Evolutionarily conserved elements in imprinted regions differ from those associated with autosomal genes in various ways. Whereas for maternally expressed genes strong divergence of protein-encoding sequences is most prominent, paternally expressed genes exhibit substantial conservation of coding and noncoding sequences. Conserved elements in imprinted regions are marked by enrichment of CpG dinucleotides and low (TpG+CpA)/(2·CpG) ratios indicate reduced CpG deamination. Interestingly, paternally and maternally expressed genes can be distinguished by differences in G+C and CpG contents that might be associated with unusual epigenetic features. Especially noncoding conserved elements of paternally expressed genes are exceptionally G+C and CpG rich. In addition, we confirmed a frequent occurrence of intronic CpG islands and observed a decelerated degeneration of ancient LINE-1 repeats. We also found a moderate enrichment of YY1 and CTCF binding sites in imprinted regions and identified several short sequence motifs in highly conserved elements that might act as additional regulatory elements.

**Conclusions:**

We discovered several novel conserved DNA features that might be related to allele-specific DNA methylation. Our results hint at reduced CpG deamination rates in imprinted regions, which affects mostly noncoding conserved elements of paternally expressed genes. Pronounced differences between maternally and paternally expressed genes imply specific modes of evolution as a result of differences in epigenetic features and a special response to selective pressure. In addition, our data support the potential role of intronic CpG islands as epigenetic key regulatory elements and suggest that evolutionary conserved LINE-1 elements fulfill regulatory functions in imprinted regions.

## Background

Imprinted genes are monoallelically expressed in a parent-of-origin way, i.e. one of the two alleles is silenced depending on its parental origin. They are often found in clusters around differentially methylated regions (DMRs) that are characterized by hypermethylated DNA on one chromosome but hypomethylated DNA on the other [1,2]. The specific DNA methylation patterns are established during germ cell development and maintained after fertilization [3-5]. In human and mouse, a steadily growing number of approximately 100 imprinted genes have been identified to date [6,7]. It is estimated that a few hundred genes may be subject to imprinting [8-10].

In order to decipher the particular epigenetic properties that distinguish imprinted genes from the majority of genes that are biallelically expressed, their DNA sequences have been intensely analyzed [11-16]. The major aim of such studies was to identify DNA sequence features that support the establishment and maintenance of allele-specific modifications. One of the most immediate findings was that repetitive elements show a particular behavior in imprinted regions: Short interspersed transposable elements (SINEs) are reduced in the vicinity of human and mouse imprinted genes whereas long ones (LINEs, especially of the L1 subfamily), long terminal repeats, simple repeats, and low complexity regions as well as tandem repeats occur more frequently. In combination with other sequence features, the distinct distribution of repetitive elements has subsequently been used to predict putative imprinted genes in the mouse and human genomes [9,10].

Imprinted gene expression in mammalian species is strongly conserved in the sense that the orthologs of most imprinted genes are also monoallelically expressed in other species. For this reason, one might expect that also DMRs, which represent the key regulatory elements in imprinted regions, exhibit a strong conservation of their DNA sequences. Interestingly, this is not the case. Instead, a common conserved feature of functionally orthologous DMRs is the presence of tandem repeats that can be composed of highly divergent motifs in the individual species [17,18]. Indeed, detailed analyses revealed that CpG islands associated with imprinted genes contain more frequently tandem repeats than the CpG islands of randomly selected genes [16]. Thus, for identification of imprinting centers rather the presence of tandem repeats than conservation of the DNA sequence appears to be a useful indicator. Nevertheless, highly conserved elements outside of genes or CpG islands have been identified in imprinted regions [17,19] and some of these elements have been shown to act as additional regulatory elements such as tissue-specific enhancers [20].

Among transcription factors and chromatin organizers that bind to specific DNA motifs, CTCF and Yin-Yang 1 (YY1) appear to play prominent roles in genomic imprinting. YY1 has been suggested to recruit histone H3K27 tri-methylase to the repressed allele of imprinted genes [21]. In line with this suggestion, conserved tandem repeats in the DMRs of *Peg3 *and at the *Gnas *locus contain YY1 binding sites [22] and also the Prader-Willi/Angelman Syndrome region is associated with YY1 binding sites [23]. Furthermore, YY1 interacts with CTCF [24], a methylation-sensitive transcription factor that was shown to inhibit the interaction of the *Igf2 *promoter with the enhancers downstream of *H19 *[25,26] by formation of chromatin loops [27]. In addition, CTCF binding sites have been identified at several other imprinted loci [28-30].

Although detailed studies have addressed CpG islands and repetitive elements, little attention has been paid to the general issue of DNA sequence conservation in imprinted regions, especially of noncoding sequences. This is surprising as monoallelic silencing of imprinted genes is a conserved mechanism of gene regulation suggesting that many of their regulatory elements might be tightly conserved. Moreover, in a recent publication we showed that the monoallelic expression of imprinted genes is associated with unusual conservation patterns of protein-coding sequences, indicating that these genes differ from biallelically expressed genes in their reaction to natural selection [31].

The function of a gene is determined by the encoded protein or noncoding RNA sequence and its temporal or tissue-specific expression pattern, which is under influence of various regulatory elements such as promoters, enhancers or silencers that reside in noncoding regions. Therefore, differences in response to natural selection between imprinted and non-imprinted genes might result in different conservation patterns of noncoding sequences. In addition, germline specific DNA methylation and histone modifications may influence mutations rates and DNA repair efficiency [32]. Taken together, these factors may result in specific patterns of sequence conservation of regulatory elements in noncoding regions of imprinted genes compared to biallelically expressed genes. Uncovering such differences is one of the important research questions of this study.

Addressing the issue of sequence conservation at imprinted loci, we have compared genomic sequences and highly conserved elements of imprinted genes to those of all autosomal genes in the human and mouse genomes. We found that they show differences in terms of length, conservation, G+C content, and CpG content. Furthermore, imprinted genes seem to be less affected by CpG deamination than other genes, indicating that differential methylation may correspond to either a relative hypomethylation or strong purifying selection at CpG positions. Enrichment of intronic CpG islands and ancient repetitive elements, particularly LINE-1 (L1), indicates that these elements constitute important functional elements of imprinting. Conserved intergenic and intronic regions are enriched in CpG-rich motifs, arguing for an open chromatin structure and possible functions as promoters of antisense or alternative transcripts. In contrast, sequence features of promoter regions suggest that the transcriptional regulation of imprinted genes on the active allele is in general similar to that of biallelically expressed genes.

## Results

### Imprinted genes are flanked by long intergenic regions

In order to get a comprehensive picture of DNA sequence properties in imprinted regions, we compared a set of 58 protein-coding imprinted genes to all 17,916 protein-coding autosomal human genes from the UCSC Genome Browser RefSeq genes track [33]. The human imprinted group consists of genes that are orthologous in human and mouse and for which imprinting has been reported in at least one of the two species in the literature. Applying the same procedure for the mouse yielded 18,772 genes on autosomes. The imprinted set in mouse excludes five orthologs that are not annotated as RefSeq genes; additionally, parental expression patterns are different for some orthologs. Information about the imprinted genes and their allele-specific expression is given in additional file 1.

Table 1 shows the sequence properties of human imprinted and autosomal genes; data for the mouse are given in Additional file 2. In the human, the G+C content is insignificantly elevated for imprinted genes compared to the level of all autosomal genes (Wilcoxon test, p > 0.1). With a median of 46%, the G+C content is essentially the same in all murine gene groups (p > 0.2). In both species, CpG content of imprinted genes as measured by the CpG_obs_/CpG_exp _ratio is insignificantly increased (p > 0.05). Although imprinted genes are not significantly longer than biallelically expressed ones, we noted differences when analyzing their exon and intron structure in more detail. A previous report described reduced intron contents of paternally expressed human and mouse genes and an enrichment of introns in maternally expressed mouse genes relative to a non-imprinted control set [34]. Here, we found that, compared to autosomal genes, especially maternally expressed genes tend to possess longer introns in human (p < 0.02), but not in the mouse (p > 0.05). In the maternally expressed group, *KLF14 *(*Klf14*) is the only intronless gene. Genes without introns in the paternally expressed group (*DIO3*, *MAGEL2*, *MKRN3*, *NAP1L5*, *NDN*) are tentatively enriched compared to the human autosomes, where there are 1136 intronless genes (Fisher's exact test, p < 0.04). On mouse autosomes, there are 2037 intronless genes, a significantly larger number than in the human (p < 0.0001). Hence, in mouse the enrichment in the paternally expressed set is not significant (p > 0.3).

**Table 1:** General sequence properties of human genes

	imprinted	maternally expressed	paternally expressed	autosomal
G+C content of genes	46.56%	46.91%	45.98%	45.47%

CpG_obs_/CpG_exp _of genes	0.305	0.305	0.315	0.280

gene length (bp)	29641	46976	26829	22422

intron length (bp)	1637*	1652	1633	1549

length of intergenic regions (bp)	50879*	49483	55322	26410

coverage of introns with purely intronic PCSs	0.74%	0.69%	1.09%	0.45%

purely intronic PCSs per 10 kb of intron per gene	1.72	1.35	2.04	1.06

coverage of intergenic regions with PCSs	0.66%	0.74%	0.64%	1.11%

intergenic PCSs per 10 kb per gene	1.29	1.41	1.29	2.17

* p < 0.01 (Wilcoxon test for comparison with autosomal genes)

Gene length: length of transcribed DNA sequence from most upstream transcriptional start site to most downstream transcriptional termination site; intergenic region: For this, the intergenic segments between any two genes were cut into two halves. Each of them was assigned to the closest gene. As intergenic region of a gene we then added the lengths of the halved upstream intergenic segment and of the halved downstream intergenic segment. Since the features show a highly skewed distribution, medians are given instead of averages and standard deviations.

In the human, intergenic regions assigned to imprinted genes (median 50 kb) are longer than on the autosomal level (Wilcoxon test, p < 0.01), however, in mouse, this size difference represents only a trend (p < 0.05 for mouse). As a consequence of longer introns and intergenic spaces, there is an increased statistical chance of encountering genomic features such as repetitive elements, CpG islands, or conserved elements in the vicinity of imprinted genes.

### Conserved elements in imprinted regions are CpG rich

Sequence conservation in protein-coding regions gives clues about structural and functional conservation of the encoded proteins. In contrast, conserved DNA segments in noncoding sequences may serve as indicators for evolutionarily conserved regulatory elements. Hence, such elements, which that can be up to several hundred base pairs long, are interesting subjects for investigations on relationships between sequence conservation and epigenetic regulation of imprinted genes. Addressing the conservation of DNA sequences on a genome-wide scale, we investigated the phastCons28wayPlacMammal most conserved sequences (PCSs) from the UCSC Genome Browser. These DNA elements are conserved among 18 eutherian mammals and have been identified through multiple sequence alignments of vertebrate genomes [35]. After mapping 1,271,956 PCSs of at least 20 bp length onto autosomal human genes, 3969 PCSs were assigned to the imprinted group. Of these, 2102 belong to the 28 maternally expressed, and 1867 to the 30 paternally expressed genes, respectively. In order to verify that obtained results are not biased by the properties of the human genome, we repeated the analyses for the mouse. From the phastCons30wayPlacMammal track we extracted 1,268,568 highly conserved elements of at least 20 bp, of which 3502 reside in the vicinity of the murine imprinted genes. In the following, we refer in most cases only to the human since we found essentially the same patterns in the mouse (Additional file 2).

Imprinted genes possess CpG-rich differentially methylated regions that are hypermethylated on one allele. Since cytosine methylation is associated with elevated accumulation of CpG to TpG transitions, epigenetic events might influence sequence conservation and result in unusual CpG contents of DNA sequences. In general, PCSs associated with imprinted genes possess higher G+C contents and higher CpG_obs_/CpG_exp _ratios compared to all autosomal PCSs (Wilcoxon test, p < 0.001). These effects are most pronounced for intronic PCSs (Table 2). Additionally, the portion of PCSs that contain at least one CpG is higher in the imprinted group than for the autosomes (42% vs. 36%, χ^2 ^test, p < 0.001).

**Table 2:** Features of different PCS classes in human

group	number of PCSs	conser-vation score	length (bp)	G+C content	PCSs with ≥ 1 CpG	CpGobs/CpGexp of PCSs with ≥ 1 CpG	(TpG+CpA)/(2·CpG) ratio of PCSs with ≥ 1 CpG
**overlapping with CpG islands**							

imprinted	321	392	69	65.93%	94.38%	0.79	0.73

maternal	170	417	75	66.48%	95.29%	0.85^a^	0.59

paternal	151	379	62	65.44%	92.72%	0.74*	0.83*

autosomal	55931	385	58	67.48%	95.30%	0.83	0.68

**intronic**							

imprinted	1120	320*	40	38.89%****	32.05%***	0.51****	2.50*****

maternal	719	320	41	39.39%****	33.38%***	0.52**	2.50*****

paternal	401	320	40	38.10%**	29.68%	0.50**	2.50****

autosomal	365258	329	40	36.54%	25.42%	0.42	3.83

**intergenic**							

imprinted	1787	325****	40****	39.04%*****	33.97%***	0.47****	2.83*****

maternal	832	320****	39***	35.78%^c^	25.00%^b^	0.53****^a^	3.00****

paternal	955	329***	41	42.31%***	41.78%***	0.45*	2.50*****

autosomal	588309	338	43	36.36%	27.32%	0.42	4.00

**unique**							

imprinted	2357	325****	39****	38.46%*****	29.87%***	0.45****	3.00*****

maternal	1304	320*****	39****	36.72%^c^	25.61%^b^	0.46****	3.00*****

paternal	1053	329***	40	40.74%*****	35.14%***	0.44	3.00*****

autosomal	844703	338	42	36.36%	25.49%	0.39	4.33

* p < 0.01, ** p < 0.005, *** p < 0.001, **** p < 1e^-4^, ***** p < 1e^-10 ^for comparison with autosomal PCSs (χ^2 ^test for fractions of PCSs, Wilcoxon test for all other features). If maternally and paternally expressed genes differ significantly from each other, this is indicated as following: ^a ^p < 0.05, ^b ^p < 0.005, ^c ^p < 1e^-4 ^

Unique PCSs do not overlap with protein encoding exons, repetitive elements, or CpG islands. The shown absolute numbers of PCSs are not normalized by the lengths of the analyzed genomic segments. Hence they give information about the amount of available data but not about frequencies or coverage of PCSs in the listed genomic segments.

We previously introduced an additional estimate of CpG deamination by the (TpG+CpA)/(2·CpG) ratio, which can be regarded as an indicator for CpG to TpG transitions rates [36]. High (TpG+CpA)/(2·CpG) values hint at an accumulation of the potential deamination products of methylated cytosines whereas low values indicate maintenance of CpGs, which might result from reduced methylation levels. In contrast to the CpG_obs_/CpG_exp _ratio, the calculation of (TpG+CpA)/(2·CpG) is independent of the G+C content. Regarding the CpG-containing PCSs in imprinted regions, the median (TpG+CpA)/(2·CpG) ratio is lower than on the autosomal level (Wilcoxon test, p < 0.001). This effect is mostly caused by lower (TpG+CpA)/(2·CpG) ratios in PCSs in intronic and intergenic regions. Detailed numbers are given in Table 2.

### CpG islands of imprinted genes show similar levels of sequence conservation as those of autosomal genes

Enrichment of CpG dinucleotides is typical for CpG islands, which are believed to be epigenetic key regulatory elements. For this study, we used the UCSC annotations of CpG islands that are close to the original criteria established by Gardiner-Garden and Frommer [37] but are based on a higher CpG content and exclude repetitive elements. Strengthening our previous findings of enrichment of intronic CpG islands in imprinted genes [16], we found that in human, 15 out of 57 imprinted genes (29.82%) and in mouse, 11 out of 53 (20.75%) possess at least one intronic CpG island that can be regarded as potential promoter for antisense transcripts. This is significantly more than the 8.50% and 3.77% for autosomal human and mouse genes, respectively (χ^2 ^test, p < 0.001).

Addressing the conservation of CpG islands, we observed that eight percent of the PCSs in human imprinted regions overlap with CpG islands whereas the autosomal ratio is only four percent (χ^2 ^test, p < 0.001). In the mouse, the values are six and three percent, respectively (p < 0.001). For both species, the enrichment is most prominent for the group of intronic PCSs. However, CpG islands in imprinted regions do not exhibit special levels of sequence conservation: 66% of the 137 human and 84% of the 64 murine CpG islands overlap with PCSs, which is similar to the autosomal rate of 68% and 86%, respectively (χ^2 ^test, p > 0.8). The percentage by which CpG islands are covered by PCSs as well as their conservation score are virtually identical for all groups (Wilcoxon test, p > 0.8). With a median of 0.69 in human and 0.65 in mouse, the (TpG+CpA)/(2·CpG) ratio of imprinted CpG islands is increased in comparison to the ratio in all autosomal CpG islands (median 0.60 for human, 0.57 for mouse; Wilcoxon test, p < 0.005). In contrast, the CpG_obs_/CpG_exp _ratio is not significantly different (p > 0.05). This discrepancy might be due to the fact that CpG islands have to exceed a certain CpG_obs_/CpG_exp _ratio threshold by definition. In summary, the CpG richness of PCSs does not coincide with stronger conservation or elevated CpG contents of CpG islands.

Interestingly, especially PCSs that overlap with CpG islands associated with paternally expressed genes have a lower G+C content and lower CpG_obs_/CpG_exp _ratio and a higher (TpG+CpA)/(2·CpG) ratio. For maternally expressed genes, we observed opposite patterns, which are however not statistically significant (Table 2). This observation suggests that maternally and paternally expressed genes may differ in their epigenetic marks.

### Conserved elements of imprinted genes overlap frequently with L1 elements

**Figure 1:**
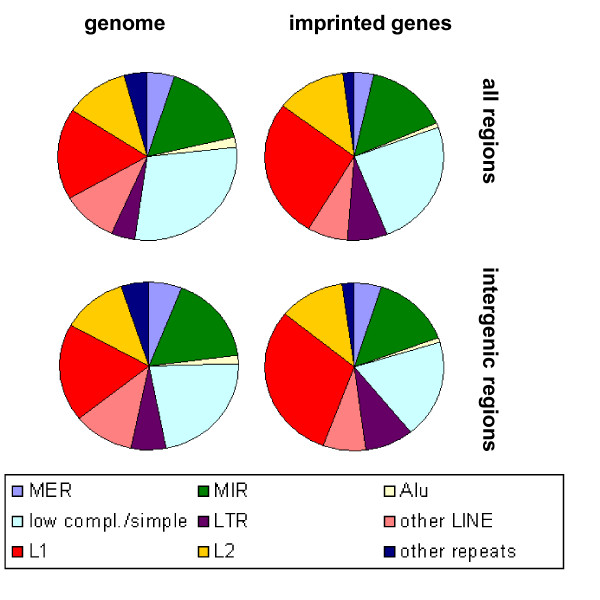
**Distribution of repetitive elements in phastCons sequences**. PCSs that contain at least 1 bp of a repetitive element were summed up in categories according to the first overlapping repeat. The most prominent enrichment is that of PCSs overlapping with LINE-1 repeats (L1, red) in the intergenic regions of imprinted genes relative to the autosomal genome. MER: medium reiteration frequency repeats; MIR: mammalian-wide interspersed repeats; LTR: long terminal repeats; low compl./simple: low complexity regions and simple repeats; L2: LINE-2 repeats.

Since different repetitive elements have been reported to be enriched or depleted in imprinted regions [11-16], we investigated whether there is also a special connection between repetitive and conserved elements. Indeed, imprinted regions possess more PCSs that overlap with repetitive elements than autosomal regions (human: 12% vs. 8%, mouse: 10% vs. 7%, χ^2 ^test, p < 0.001). The enrichment is highly significant for both intergenic and intronic regions in both species. As figure 1 shows, repeat-containing PCSs in imprinted regions of both human and mouse overlap significantly more often with LINEs compared to autosomes (χ^2 ^test, p < 0.005). It has been suggested before that imprinted regions are enriched in L1 repeats [15]. Our data show that the coverage with L1 repeats is tentatively elevated in imprinted intergenic regions (median 14%) compared to autosomal intergenic regions (median 11%; Wilcoxon test, p < 0.02), whereas in intronic regions it is about 6% in all groups. However, the percentage of L1 elements that contain PCSs (and therefore can be regarded as conserved) is not significantly elevated in imprinted compared to autosomal regions (1.98% vs. 1.77%, χ^2 ^test, p > 0.1). Furthermore, conservation scores and lengths of these PCSs and lengths of the associated L1 repeats do not differ significantly (Wilcoxon test, p > 0.3). Thus, the enrichment of PCSs that reside in L1 repeats seems to result from combination of subtle effects, i.e. a slightly higher coverage and marginally elevated conservation of L1 in imprinted regions.

The 130 PCSs of imprinted genes that overlap with L1 elements do not show distinctive features in terms of G+C and CpG content. Nevertheless, when the entire sequences of intergenic L1 elements were investigated, for the imprinted set we observed an elevation of their CpG_obs_/CpG_exp _ratio (median 0.14 vs. 0.13; Wilcoxon test, p < 0.0002) and their (TpG+CpA)/(2·CpG) ratio is significantly reduced (median 12.00 vs. 12.70; p < 0.0003), indicating a rather mild loss of CpGs. This is of particular interest when regarding their age distribution: 81% of these L1 elements belong to the ancient L1 M subgroup whose origin predates the mammalian radiation whereas in autosomal intergenic regions, only 76% of the L1 elements belong to the L1 M subgroup (χ^2 ^test, p < 0.001).

Corresponding to the previously reported depletion of SINE elements [11,16], PCSs overlapping with SINEs are reduced in murine imprinted regions (χ^2 ^test, p < 0.001) but not in human (p > 0.1). PCSs that overlap with other types of repetitive elements do not show significant differences.

### Divergence of protein-encoding exons of maternally expressed genes

**Figure 2:**
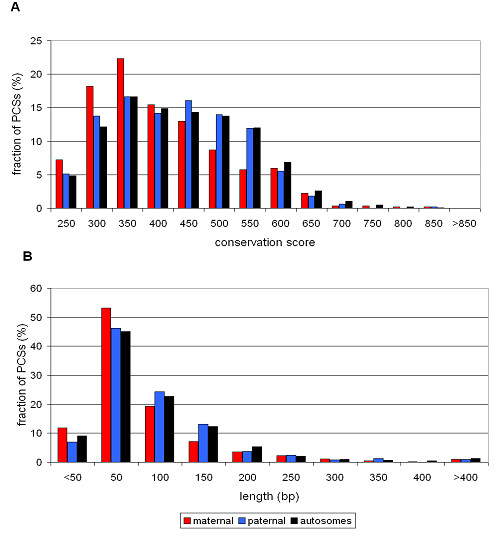
**Conservation score and length of exonic phastCons sequences**. Using the human genome as a reference, conservation scores (A) and lengths (B) of PCSs that overlap with coding exons were determined. Compared to autosomal data (black bars), the PCSs of maternally expressed genes (red bars) are shorter and have lower conservation scores whereas PCSs of paternally expressed ones (blue bars) are similar to PCSs of autosomal genes.

In our previous work we have shown that especially maternally expressed genes are prone to reduced conservation of protein encoding sequences [31]. These observations on cDNA and protein level were confirmed here by PCSs: The 1024 PCSs overlapping with coding exons of imprinted genes are significantly shorter and have lower conservation scores than all 309,941 coding PCSs as well as randomly sampled groups of 1024 PCSs in coding regions of other autosomal genes (Wilcoxon test, p < 0.0002). A closer investigation as shown in figure 2 revealed that these differences are caused by the subset of 538 coding PCSs of the 28 genes with maternal expression in human.

The coding parts of exons are of similar length in imprinted and autosomal genes (Wilcoxon test, p > 0.8). Interestingly, those of maternally expressed genes (median 131 bp) tend to be longer (p < 0.02) and those of paternally expressed ones (median 111 bp) are shorter (p < 0.007) than those of the autosomes (median 125 bp). Thus, shorter exons are not responsible for a decreased length of PCSs in maternally expressed genes. The proportions by which PCSs overlap with coding exons are even higher for imprinted genes compared to the rate for all protein-coding human genes (Wilcoxon test, p < 0.0001).

In order to differentiate between the contribution of protein-coding sequences and adjacent intronic parts to PCSs, we separately investigated the subsets of PCSs that are completely located in coding exons. They comprise 51% of those in the imprinted group and 41% of the autosomal ones (χ^2 ^test, p < 0.001). Here, the weak conservation of PCSs in all imprinted genes and in maternally expressed genes was less significant (Wilcoxon test, p < 0.02) and the lengths became similar (p > 0.05). In contrast, PCS that only partially overlap with coding exons are significantly shorter and less conserved, especially in maternally expressed genes (p < 0.0002). Together with the increased exon overlap rate, this implies that intronic sequences near exon boundaries contribute substantially to the differences between PCSs in coding exons of imprinted genes and those of biallelically expressed genes.

### Paternally and maternally expressed genes show different conservation patterns of noncoding sequences

**Figure 3:**
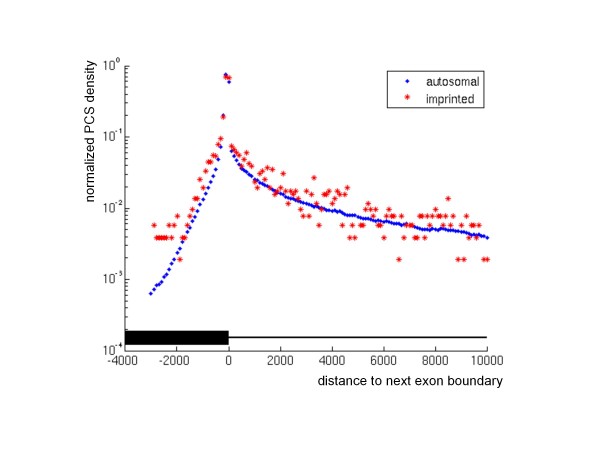
**Distribution of phastCons sequences at exon boundaries**. Shown are the frequencies of PCSs in introns and exons as a function of their distance from the closest exon boundary in a semi-logarithmic representation. The y-axis shows the normalized PCS density calculated as the number of PCSs per 100 bp bin divided by the number of introns in the respective group (imprinted, autosomal). On the x-axis, the position of the exon boundary is marked as zero. PCSs with negative distances are either partially or completely located in exons (thick black bar). Most of these are concentrated within a distance of 2000 bp from the exon boundary. Likewise, the number of PCSs that are completely located in introns (thin black line; positive distances) decreases rapidly within increasing distance. Beyond a distance of 10 kb (not shown here), only a few PCSs are found. Imprinted genes (red stars) possess more PCSs within about ± 2000 bp of the exon boundary than autosomal genes (blue diamonds).

Besides protein-encoding sequences, also noncoding DNA elements may substantially influence the functions of genes. Among such noncoding elements, splice donor and acceptor sequences that are found at exon-intron boundaries are prominent examples. For this reason, we analyzed intronic PCSs, especially those in the vicinity of exons, in more detail. As the slightly increased intron length observed for imprinted genes might influence their PCS content, we normalized the number of PCSs by the length of the respective regions. The resulting coverage by intronic PCSs in imprinted genes is tentatively higher compared to all autosomal genes (Wilcoxon test, for human: p < 0.05; for mouse: p < 0.003; Table 1, Additional file 2). Remarkably, we found that the number of PCSs decreases dramatically with their distance from the next exon (Figure 3). In close distance of up to 1 kb from the next exon, the density of intronic PCSs is slightly higher for paternally expressed genes (median 2.70% as opposed to 1.61% for autosomal genes; Wilcoxon test, p < 0.05) whereas this is not the case for maternally expressed genes (median 1.39%, p > 0.5). Hence, the conservation of the protein-coding portions of paternally expressed genes is accompanied by substantial conservation in the neighborhood of exon-intron boundaries. As for all PCSs, we observed increased G+C and CpG contents for PCSs located in introns of imprinted genes (Table 2).

Within mature mRNAs, conserved elements in untranslated regions (UTRs) of the mRNAs might act as regulatory elements on pre- and post-transcriptional level. Such elements may be important for RNA stability. Interestingly, the UTRs of imprinted genes seem to be only marginally conserved: Among 6537 PCSs in UTRs, only five belong to imprinted genes (χ^2 ^test, p < 0.001). This low number makes a detailed statistical analysis of these PCSs impossible.

Outside of transcribed regions, conserved elements might influence the promoter activities of nearby genes. After normalization by sequence length, we observed a slightly reduced coverage with PCSs in intergenic regions (Table 1, Additional file 2), but the differences did not reach statistical significance (p > 0.05). Here, all groups showed a highly similar pattern of decreasing PCS content with increasing distance from the next gene (data not shown). In general, gene distance is uncorrelated with conservation score or length of the PCSs (Pearson's r < 0.06) and PCSs in different distance windows do not show consistent differences.

Interestingly, intergenic PCSs assigned to paternally and maternally expressed genes, respectively, differ in terms of their sequence feature from each other: The latter are shorter and have a lower conservation score and G+C content, and a higher CpG_obs_/CpG_exp _ratio (Table 2).

Regulatory elements outside of promoter regions are assumed to reside only rarely in exons or repetitive elements. Hence, a possibly function of conserved elements outside of exons, repetitive elements, or CpG island promoters might be regulatory enhancer or silencer functions. In order to address differences in conservation of DNA sequences, G+C and CpG contents of such elements we formed an own class of unique PCSs that do not overlap with exons, repetitive elements, or CpG islands. Also these unique PCS elements show distinguishing features for imprinted genes (Table 2). Elevated G+C content and presence of at least one CpG are characteristic for PCSs assigned to paternally, but not maternally expressed genes. Unique PCSs of both maternally and paternally expressed genes are shorter and possess decreased conservation scores in comparison to unique PCS associated with autosomal genes.

### Conservation of short CpG-rich sequence motifs

Promoters contain transcription factor binding sites that directly mediate gene expression. Detailed analyses of transcription factor binding sites in the promoter regions of imprinted genes have been reported elsewhere [38]. Therefore, we focused on general sequence patterns. In the promoter region defined as the sequences from -1000 to the most upstream transcriptional start site, 67% of the imprinted genes and 61% of the autosomal ones have at least one PCS (χ^2 ^test, p > 0.4). Also in terms of general sequence features such as G+C content, CpG_obs_/CpG_exp _and (TpG+CpA)/(2·CpG) ratios, overlap with CpG islands, and conservation scores of PCSs, promoters of imprinted genes are highly similar to those of all autosomal genes (data not shown).

We next aimed at identifying short sequence motifs that are overrepresented in promoters of imprinted genes compared to both the genomic background and promoters of autosomal genes. Using the program *K-Factor *[39], we detected two 6 bp motifs with a significant enrichment (*K-Factor *score ≥ 3.5) in the regions 1000 bp upstream of the transcriptional start site in human imprinted genes (tgcgta and gcgtat) and seven different ones in mouse imprinted genes (atagcg, atcgca, cgtacg, ctacga, tgcgtg, tgtcga, ttggcg). Indicating their association with CpG islands, all of these motifs share the feature of having a CpG dinucleotide. Furthermore, the occurrence of TpG hints at possible effects of deamination. When scanning the motifs with the *TransFac *tool *Match *[40] we found that two murine motifs correspond well to known transcription factor binding sites, namely CCAAT box (ttggcg) and AhR/Arnt (tgcgtg).

With *K-Factor *we also identified a large number of motifs that are overrepresented in intronic PCSs in the imprinted set. All of them contain at least one CpG whereas TpG and CpA are rare, which is in accordance with the lowered (TpG+CpA/(2·CpG) ratios of intronic PCSs. Table 3 shows the ten 6-mers that show a significant enrichment in both human and mouse imprinted sets compared to both the pre-calculated genomic background and the autosomal intronic PCSs. Similar motifs were detected for intergenic PCSs (data not shown) but only one of them, cgtcga, is overrepresented in both human and mouse intergenic regions of imprinted genes. In general, there were no genome-wide overrepresented 6-mers that were underrepresented in the imprinted groups. Interestingly, the motifs identified in the intronic PCSs contain G+C and CpG more frequently than the elements identified in the most upstream transcriptional start sites. For the intergenic and intronic motifs from Table 3, *Match *revealed the possible transcription factors ATF, v-Myb and the EBV transcription factor R.

**Table 3:** 6-mers enriched in intronic PCSs of imprinted genes

	human imprinted		mouse imprinted		
**6-mer**	**score against genomic background**	**score against autosomal PCSs**	**score against genomic background**	**score against autosomal PCSs**	**matching transcription factor(s)**

**cg**c**cg**c	6.21	4.08	5.91	4.04	

**cgcg**ac	3.51	4.31	3.64	3.82	

gc**cgcg**	5.79	4.47	5.46	4.39	

gc**cg**tc	3.62	4.32	5.37	4.70	ATF, v-Myb

g**cg**c**cg**	6.02	3.65	9.36	5.92	

g**cg**t**cg**	4.31	6.65	5.96	6.52	ATF

gggc**cg**	3.82	4.01	3.70	3.99	

gggg**cg**	5.30	4.28	4.75	3.61	R

gt**cgcg**	10.53	7.32	10.92	5.60	

tc**cgcg**	4.51	4.18	4.68	3.96	

### CTCF and YY1 binding sites are only slightly enriched in imprinted genes

As CTCF and YY1 are supposed to act as regulators of imprinted genes [22-26], we analyzed the association of imprinted genes with potential binding sites for these factors in more detail. Focusing on a set of CTCF binding sites that were identified in an unbiased genome-wide analysis [41], we found CTCF binding sites in the introns of 20 imprinted genes (34.48%), which is a slight enrichment compared to 21.65% of the autosomal genes (χ^2 ^test, p < 0.05). With regard to intergenic regions, 17 imprinted genes (29.31%) and 4734 autosomal genes (26.42%) have a nearby CTCF binding site (p > 0.8). In total, CTCF binding sites are present within or in the vicinity of 55.17% of the human imprinted genes and 40.96% of the autosomal ones (p < 0.05). When requiring these sites to overlap with PCSs, the numbers drop considerably: Only 16 imprinted and 4290 autosomal genes are associated with a conserved CTCF binding site (23.95% or 27.59%, respectively; χ^2 ^test, p > 0.6). Hence, CTCF binding sites are apparently not part of highly conserved regulatory modules.

Predicted YY1 binding sites that are conserved between human, mouse, and rat are found in introns of 16 human imprinted genes, including both previously reported genes and new ones, and 3347 autosomal genes (27.59% vs. 18.68%, p > 0.1). In intergenic regions, 20 imprinted genes (34.48%) possess a YY1 binding site compared to the autosomes with 23.58% (p > 0.05). If all locations are taken into account, the ratio increases to 53.45% for imprinted and 35.52% for autosomal genes (p < 0.01). Since CTCF and YY1 interact physically [24], a combined occurrence of binding sites for both proteins might be particularly meaningful. This is the case for 34.48% of the human imprinted genes as opposed to 19.66% on autosomes (p < 0.01). However, the unchanged p value indicates that the combination of both binding sites did not result in an increased enrichment. Hence, the co-occurrence of both binding sites is apparently not a prominent feature of imprinted gene regulation.

## Discussion

In this study, we have identified highly conserved DNA elements in imprinted genes and compared them to all autosomal genes in the human and mouse genomes. We observed some characteristic features that appear to be related to their allele-specific DNA methylation. Analyses of general sequence features confirm previous data such as longer introns, enrichment of intronic CpG islands, depletion of SINE repeats, and enrichment of LINE repeats [11-16,34,42]. Moreover, imprinted genes are more distant from their neighboring genes. They differ in conservation of protein-encoding and noncoding sequences from autosomal genes. In addition, short sequences that are highly conserved in mammals (PCSs) show differences in the accumulation of CpG mutations that relate sequence conservation to epigenetic features such as DNA methylation. Lastly, paternally and maternally expressed genes can be distinguished by their sequence conservation patterns in coding and noncoding sequences. It should be noted that features such as G+C and CpG content and repetitive elements correlate or anticorrelate with each other. For example, the LINE-1 content is higher in G+C poor sequences than in G+C rich sequences [43]. Hence, an interesting topic of future research might be an in-depth analysis of interactions between different sequence features in imprinted regions.

PCSs in imprinted regions not only show elevated G+C and CpG contents and increased overlap with CpG islands but also a reduced (TpG+CpA)/(2·CpG) ratio, which can be regarded as an indicator for low C to T transitions rates [36]. This effect is, however, not associated with a stronger conservation of CpG islands. Instead, CpG islands of imprinted genes are characterized by elevated (TpG+CpA)/(2·CpG) ratios. This suggests that their methylation levels in the germline and subsequent deamination rates might be higher than those of CpG islands of normal autosomal genes, which are usually unmethylated. In contrast, high G+C and CpG contents outside of CpG islands might result from hypomethylation compared to autosomal genes. Such a scenario is reminiscent of observations for the inactive X chromosome in females that is hypermethylated only at CpG islands but hypomethylated in regions outside of these regulatory elements [44]. Therefore, our observations may indicate allele specific or germline specific DNA methylation outside of the known DMRs of imprinted genes. An alternative explanation for the elevated CpG content might be the need of a certain CpG density in these elements that allows establishment and maintenance of methylation marks.

Our results are stable towards the inclusion of additional imprinted genes: After addition of five new imprinted genes, the p values were found to be stable or even smaller for most differences, except for those of the lengths of intergenic and unique PCSs. Additional analyses based on randomly sampled gene sets confirmed that significant differences between imprinted and autosomal genes are indicated by p values below 0.005 whereas trends with higher p values have to be interpreted with caution (data not shown).

In a previous study, we concluded that the increased divergence of maternally expressed genes occurred most likely due to reduced selective pressure [31]. Here, we show that paternally expressed genes, which are in terms of sequence conservation highly similar to all autosomal genes, have a slightly higher coverage with intronic PCSs. As this effect is most pronounced in exon-near regions, the strict conservation of paternally expressed protein-encoding sequences might be supported by conservation of splice signals. Most interestingly, some classes of PCSs exhibit pronounced differences in terms of G+C and CpG content between maternally and paternally expressed genes. The high CpG content of unique PCSs of paternally expressed genes, however, is not associated with elevated conservation scores. Hence, a conventional conservation of regulatory elements that would coincide with the strong conservation on protein level is probably not the driving force. Lastly, in the parental germlines, paternally and maternally expressed genes might acquire different, temporal methylation marks outside of known DMRs. Such transient marks may later be removed by epigenetic reprogramming processes. Due to different mutation rates and repair efficiencies, differential methylation might result in differences in the evolutionary retention of CpGs in paternally and maternally expressed genes.

Conserved elements in imprinted regions overlap with repetitive elements more frequently than those of all autosomal genes. Taking into account that most SINEs are either primate or rodent specific, it is not surprising that the overlap of PCSs with SINEs in imprinted regions is similarly low as that of the whole autosomal genome. In contrast, more PCSs overlap with L1 elements and these PCSs show elevated conservation scores. Interestingly, the proportion of L1 elements that belong to older classes of repetitive elements is elevated at imprinted loci. However, a rather low number of PCSs overlap with L1 elements. Therefore it is not clear if the elevated level of ancient L1 elements is solely due to stronger conservation, or if increased integration rates in early mammals might have been its major cause as suggested by other studies [45].

We also evaluated putative transcription factor binding sites in imprinted regions. Imprinted genes exhibit a pronounced divergence in terms of their tissue-specific expression patterns [38]. Thus, it is not surprising that among the overrepresented 6mer motifs in promoters we found only one motif, tgcgtg, which is identical to the consensus sequence of a transcription factor binding site, namely the aryl hydrocarbon receptor nuclear translocator (ARNT), which is also known as hypoxia-inducible factor 1-beta (HIF1-β). A causative linkage between placental hypoxia, pre-eclampsia and misregulation of imprinted genes has been suggested [46,47]. Hence, ARNT binding sites might be an indicator for placental or embryonic key function of a number of imprinted genes. The fact that the pattern is only overrepresented in the mouse may be related to different placenta morphologies in human and mouse [48]. Our analyses confirm an enrichment of putative conserved YY1 binding sites in imprinted regions whereas experimentally validated CTCF binding sites are only moderately enriched and rarely contain PCSs. By analyzing DNA sequences of PCSs, we identified some 6mer motifs that are overrepresented in both human and mouse imprinted genes and characterized by their high CpG content. Interestingly, especially in intronic PCSs there are C+G rich motifs with similarity to GC boxes, and motifs that are similar to the binding sites of activating transcription factors (ATFs). In line with the enrichment of intronic CpG islands, such sequence motifs may indicate promoter elements for alternative or antisense transcripts that are frequent features of imprinted genes [42].

## Conclusions

In summary, we discovered pronounced differences in the conservation patterns of imprinted and autosomal genes. Changes in CpG densities and evidence for reduced CpG deamination suggest that imprinted genes differ in their DNA methylation patterns from biallelically expressed, not only at previously identified DMRs but also in coding regions, CpG islands and repetitive elements.

## Methods

### Gene selection and processing

From the Otago Catalogue of Imprinted Genes [6] and the literature we selected 58 genes for which imprinting effects have been observed at least in one species and for which orthologous sequences of human and mouse could be localized with the UCSC Genome Browser [33] for human hg18 (NCBI build 36.1, March 2006 assembly), and mouse mm9 (NCBI build 37.1, July 2007 assembly). These genes were compared to all RefSeq genes that are located on autosomes. If there were several transcripts for one gene, we took the most 5' annotated transcriptional start site and the most 3' annotated transcriptional termination site to construct the longest possible transcript. Similarly, splice variants and overlapping exons were merged in a way that the largest possible coding regions could be constructed. The genomic sequence that was assigned to a gene contained the transcribed sequence and intergenic regions upstream and downstream of the transcription unit. For determining the intergenic region, the DNA sequence between two genes was cut into two halves, each half was assigned to the nearest gene.

### Analysis of highly conserved elements

As a set of sequences with high conservation in eutherian mammals, we used the UCSC phastCons28wayPlacMammal most conserved sequences (PCSs). Such highly conserved regions were originally identified from a genome-wide multiple alignment of 29 vertebrate species by the *Phast *program [35] and afterwards projected onto a reference genome. The PCSs analyzed here are a subset of these regions showing conservation in 18 eutherian mammals. We assigned them to the longest possible RefSeq transcripts based on the human genome March 2006 assembly (hg18). It may happen that most of the conserved region is absent in human, leaving it anchored to one or a few bases followed by a gap region in the human genome compared to other genomes. Thus, we excluded elements that comprise less than 20 bp in the human genome, thereby reducing the number of PCSs by one third to 1,271,956.

The phastCons30wayPlacMammal most conserved sequences based on the mm9 mouse assembly were analyzed likewise. G+C content, CpG_obs_/CpG_exp _as a measure for normalized CpG content, and the (TpG+CpA)/(2·CpG) ratio were calculated for the according human or mouse sequences, respectively. A PCS that resides between transcriptional start site and transcriptional termination site of the respective reference gene was termed intronic if it did not overlap with an exon and coding if it overlapped by at least one base pair with a coding exon. Intergenic PCSs are located between genes and were assigned to the nearest gene.

Using a local installation of the UCSC hg18 and mm9 databases and the bioinformatics tools collection from UCSC, we searched for overlaps of genomic regions and PCSs and transcription factor binding sites that are conserved between human, mouse, and rat (tfbsConsSites). Additionally, using annotations for UCSC we identified overlaps with CpG islands and repetitive elements. We also identified overlaps with experimentally validated CTCF binding sites [41]. In order to possess a certain feature, a PCS had to overlap with its annotation by at least 1 bp.

### Statistical analysis

We performed χ^2 ^tests or Fisher's exact tests to assess whether proportions of features (e.g. relative numbers of PCSs) in the imprinted, maternally expressed or paternally expressed group were significantly higher or lower compared to those in the autosomal group. Wilcoxon tests were applied to test whether the distribution of features (e.g. length of PCSs) differed. Since differing lengths of genomic regions influence the content of PCSs, we divided their number by the summed length of the analyzed sequences per gene. We report raw p values. With a Bonferroni correction for multiple testing, a feature would be highly significant if p < 0.005. However, we also consider p values between 0.01 and 0.005 as moderately significant, and we refer to 0.01<p < 0.05 as indicating a trend.

To test whether including additional genes in the analysis would lead to substantial changes of the results, we repeated the analyses shown in Table 1 and Table 2 including five additional imprinted genes (*BLCAP*, *DLGAP2*, *PRIM2A*, *TFPI2*, and *ZNF597*). In order to investigate possible background effects, we compared randomly selected sets of autosomal genes of the same size as the imprinted set (i.e. 58 genes) with that of all autosomal genes. For each feature shown in Table 1 and Table 2, 100 such comparisons were performed. Based on this, we then counted how often randomly selected gene sets reached the same level of significance in the Wilcoxon tests as the imprinted genes.

### Motif search

For investigating the enrichment of sequence motifs, we used *K-Factor *[39] with default settings and custom Perl scripts. Sequences of PCSs in intronic or intergenic regions, respectively, were concatenated per gene, separated by 6 Ns each to prevent artificial sequence combinations. Converting repetitive elements to Ns to exclude potential motifs in repeats did not alter the motifs and only marginally influenced their scores. Possible transcription factor binding sites from the *TransFac *database were identified with the *Match *tool [40] using high quality matrices for vertebrate species, the "best selection" profile, matrix similarity = 0.7 and core similarity = 0.75. Since the original 6mers were too short to produce hits, we added five Ns to each end.

## Abbreviations

CpG_obs_/CpG_exp _ratio: ratio of observed number of CpGs to expected number of CpGs; DMR: differentially methylated region; LINE: long interspersed transposable element; L1: LINE-1; PCS: phastCons28wayPlacMammal most conserved sequence; SINE: short interspersed transposable element; YY1: transcription factor Yin-Yang 1

## Authors' contributions

BH organized the study, acquired the data, performed the statistical analyses, and drafted the manuscript. MB managed the UCSC database and wrote Perl scripts for calculating overlaps. VH participated in the interpretation of the data and revised the manuscript. MP initiated the study, contributed to its design and to the interpretation, and edited the manuscript. All authors read and approved the final manuscript.

## Supplementary Material

Additional file 1 **Imprinted genes according to RefSeq annotation**. This Excel spreadsheet gives information about the imprinted genes analyzed in this study, their parental expression, and their imprinting status in human and mouse. Click here for file

Additional file 2 **General sequence properties of murine genes**. This pdf file contains data based on mouse as in Table 1. Click here for file
