# Supplementary material for: Imprinted genes show unique patterns of sequence conservation
Source: BMC Genomics. 2010 Nov 22;11:649. doi: 10.1186/1471-2164-11-649 (PMC3091771; doi:10.1186/1471-2164-11-649)
Supplement: Additional file 2 — General sequence properties of murine genes. This pdf file contains data based on mouse as in Table 1. [file 1471-2164-11-649-S2.PDF]

General sequence properties of murine genes

	imprinted	maternally expressed	paternally expressed	autosomal
**G+C content of genes**	45.73%	45.73%	45.65%	45.89%
**CpG_{obs}/CpG_{exp} of genes**	0.24	0.22	0.32*	0.23
**gene length (bp)**	31058	44300	23787	16242
**intron length (bp)**	1262	1266	1262	1327
**length of intergenic regions (bp)**	36310	38969	28789	22296
**coverage of introns with purely intronic PCSs**	0.99%**	0.83%	1.79%	0.57%
**purely intronic PCSs per 10 kb of intron per gene**	2.02**	1.84	2.80*	1.26
**coverage of intergenic regions with PCSs**	1.25%	1.25%	1.18%	1.41%
**intergenic PCSs per 10 kb per gene**	1.93	1.54	1.99	2.46

Gene length: transcribed DNA sequence from most upstream transcriptional start site to most downstream transcriptional termination site; intergenic region: Sum of sequences upstream and downstream of a gene. For this, the distance between two genes was cut into two halves, each of these was assigned to the nearest gene.

Since features show a highly skewed distribution, medians are given instead of averages.

* $p<0.01$, ** $p<0.005$ for comparison with autosomes.
